# Annotations

**Published:** 1910-02-05

**Authors:** 


### ANNOTATIONS.

### The Increase of Exemptions.

In his annual report for last year the Chief Medical Officer of the Local Government Board gives some figures with regard to the working of the Vaccination Act of 1907. These show that the Act has been responsible for a large—and disquieting—increase in the number of exemptions. There is a reduction from 73.4 to 70.9 in the proportion of vaccinated infants, and an increase of from 5.8 to 8.4 in the number of exemptions under the conscience clauses. A very serious state of affairs is illustrated by the figures for total percentage of unvaccinated children. In 1907 this was 11 per cent. of all registered births; now it is more than 12 per cent. In 1902, 33,759 exemption certificates were granted; in 1907 the number had risen to 76,709; last year it was probably nearer 80,000. These figures are dispiriting enough to those who believe that exemption orders are a surrender to a mistaken and false spirit of toleration, which in the long run must be most detrimental to the welfare of the community. The evil effects of allowing a greater laxity in respect to vaccination than other civilised communities care to permit are well shown by Dr. Sandwith in the Gresham Lectures lately delivered. It can hardly be alleged that with such a percentage of the population unvaccinated, as these figures show to be the case, the prophylactic safeguards against an epidemic of one of the worst and most miserable diseases that afflicts mankind can by any stretch of argument be regarded as efficient.

### Mosquitoes and Leprosy.

Dr. Walter R. Brinckerhoff, Director of the Leprosy Investigation Station at Honolulu, Hawaii, has recently been making some investigations into the question as to whether the mosquito might, in any circumstances, act as a trasmitter of leprosy. Hutchinson once discussed the matter, but dismissed the theory as untenable. Dr. Brinckerhoff, after carefully reviewing the pros and cons of the subject, concludes that even if it be established that a mechanism exists whereby the mosquito can transmit lepra bacilli to a healthy person and deposit them on the skin in such a position that they will be mechanically inoculated into the tissues, it still remains a question whether the insect plays a *rôle* in the actual dissemination of the disease. The only feasible mode of transmission discussed by Dr. Brinckerhoff is that the mosquito permits the bacilli to pass through its intestinal tract without impairment of its virulence and deposits the bacilli at the time of a subsequent bite upon the skin of a healthy person. The fact that the female *Culex pipiens* does defæcate at the time of biting, according to Banks and others, makes it within the realms of possibility that the insect may act as a carrier of leper bacilli; but the data at present available do not permit of a positive statement that the mosquito acts in any respect as an agent in the transmission of leprosy, and the probabilities are against such a conclusion. Biting insects play direct and indirect parts in the spread of many diseases, but in the case of leprosy, up to the present time proof in this direction is wholly lacking.

### A Question of Courtesy.

Nearly fifty years ago the British Medical Association passed certain resolutions at a memorable meeting held at Brighton. These resolutions condemned, in no measured terms, the principles and morals of a section of the profession. It is an excellent testimonial to the broadmindedness and tolerance of the medical profession in this country that very few doctors know that such resolutions have ever been passed, and that still fewer refrain from expressing their astonishment that such resolutions were ever tabled and adopted. We have moved with the times, and it is no longer regarded as an insult for a consultant to be asked to meet a homœopath in consultation. There are many homœopaths who are members of the British Medical Association, and we venture to think that an endeavour to reiterate the sentiments expressed in the Brighton resolutions will be foredoomed to failure even at a divisional meeting. The profession, as most practitioners have found, is wide enough for all sections to dwell in amicably and peacefully, however much individuals may differ on broad principles of treatment. Nevertheless it is a fact that there flourish, here and there, hide-bound members of the medical profession whose ethical spirit is less tolerant than that of the most rabid galenist, and whose professional courtesy ceases to exist where homœopathists are concerned. We have recently come across one of this minority, and we have no desire to meet with his similars in future. This practitioner was called in to attend a patient of a brother practitioner who happens to be a homœopathist. He attended to the case but failed to notify his neighbour of the event, and left it to friends and relatives of the patient to acquaint their family practitioner with the fact that, in the latter's absence, he had been called in. The homœopath wrote a courteous note, as is usual in such cases, and requested particulars of the case. To this his colleague replied by stating that he had omitted to inform him of the case since he (the writer) was convinced that the ordinary rules of medical etiquette did not apply to homœopathists! The amazing effrontery of this statement needs no comment, but it is saddening to note that the spirit of intolerance which refuses the ordinary courtesies of professional life to a colleague on account of the fact that that colleague holds opinions which are not held by the majority of the profession, still exists in some quarters. No words can be strong enough to condemn that spirit, and it may well be asked whether a practitioner who shows himself so virulently animated by it is worthy of the professional courtesies which he refuses to extend to homœopathists.

